# Factors Affecting Ulcerative Colitis Flare-Ups: Associations With Smoking Habits and Other Patient Characteristics

**DOI:** 10.7759/cureus.19834

**Published:** 2021-11-23

**Authors:** Nadim H Malibary, Mohammed A Ezzat, Ahmed M Mogharbel, Khalid A Kouzaba, Abdulaziz A Alkadi, Usama H Malki, Sultan M Gharib, Faisal M Altowairqi, Omar I Saadah, Mahmoud H Mosli

**Affiliations:** 1 Visceral and General Surgery, Hautepierre Hospital, Strasbourg, FRA; 2 Surgery, King Abdulaziz University Hospital, Jeddah, SAU; 3 Medicine, King Abdulaziz University Hospital, Jeddah, SAU; 4 Pediatric Gastroenterology, King Abdulaziz University Hospital, Jeddah, SAU; 5 Gastroenterology, King Abdulaziz University Hospital, Jeddah, SAU

**Keywords:** saudi arabia, risk, flares, ulcerative colitis, smoking

## Abstract

Background and study aims

Currently, there are no studies conducted in the Kingdom of Saudi Arabia (KSA) that have assessed the relationship between ulcerative colitis (UC) flare-ups and smoking. The present study aims to assess the risk of UC flare-ups and evaluate the relationship between UC flare-ups and smoking in adult patients following up at King Abdulaziz University Hospital in Jeddah, KSA.

Patients and methods

This was a retrospective study involving patients with confirmed UC between January 2015 and December 2020. Various information was examined, including demographic, clinical, endoscopic, radiologic, and laboratory data. Descriptive statistics were used for summarizing findings and a logistic regression analysis was applied to test for possible associations.

Results

Eighty-nine patients with UC were included in the study. Almost half (48.3%) had recurrent UC flare-ups during follow-up. A non-significant relationship was found between recurrent UC flares and all types of smoking habits (cigarette smoking, *P *= 0.15; shisha smoking, *P *= 0.88; and vape smoking, *P *= 0.09). Participants who were underweight (*P *= 0.041), had family history of UC (*P *= 0.013), depression (*P *= 0.033), fecal incontinence (*P *= 0.003), iron deficiency anemia (*P *= 0.009), or a malignancy (*P *= 0.039) had a significantly higher probability of experiencing recurrent flares. Binary logistic regressions revealed that family history of UC (OR = 5.3, *P *= 0.007) and fecal incontinence (OR = 4.7, *P *= 0.006) were associated significantly with recurrent flares.

Conclusion

There was no clear association between smoking and recurrent UC flares identified in this cohort. Of the variables considered, UC patients with fecal incontinence or family history of UC were at the highest risk of developing recurrent flares.

## Introduction

The incidence and prevalence of inflammatory bowel disease (IBD) are increasing regionally and globally, in both developed and developing countries [1-3]. The two main forms of IBD, Crohn’s disease (CD) and ulcerative colitis (UC) can cause inflammation in the gastrointestinal tract with various clinical characteristics [4]. The exact etiology of IBD is unknown, although a multifactorial etiology has been suggested, including genetic predisposition, epithelial barrier defects, immune dysregulation, and environmental exposure [5]. UC is a chronic inflammatory disease that affects the colonic mucosa, with a tendency to involve the rectum, spreading from the distal to the proximal colonic segments in a continuous fashion, while CD can cause inflammation in any part of the gastrointestinal tract from the mouth to the anus [6]. The principal symptom of UC is bloody diarrhea, but other symptoms can occur relative to the severity and extent of the disease, such as abdominal pain, fecal urgency, and tenesmus [7]. Extra-intestinal manifestations including musculoskeletal, dermatological, and ocular can also be present and can precede or accompany gastrointestinal symptoms [8]. A diagnosis of IBD is based on clinical, radiological, endoscopic, and histological features [4].

Cigarette smoking has been linked to a less active and quiescent UC disease course [9,10]. A nationwide population-based study carried out in Japan found that the risk of developing UC was significantly higher in former smokers, and lower in individuals currently smoking [11]. Another study found that smoking patients had a milder disease compared to non-smokers, and those who quit smoking were more susceptible to frequent CD relapses [9]. Similar findings were reported in a study from the Netherlands, which showed a greater frequency of flare-ups in ex-smokers and those that had never smoked than current smokers [10]. The prevalence of smokers who initiate smoking at ≥15 years in the general population of Saudi Arabia has been reported to be 20-50% [12]. To the best of our knowledge, there are no studies in this region (the Arabian Peninsula) that have examined the potential influence of smoking habits on disease behavior in patients with UC.

This study aims to determine the proportion of UC patients with recurrent flares and to identify possible risk factors.

## Materials and methods

Study design and participants

A retrospective study was conducted on all patients with a confirmed diagnosis of UC following up at King Abdulaziz University Hospital (KAUH) in Jeddah, KSA. The KAUH inflammatory bowel disease information system (IBDIS) database was searched for adult patients (>18 years) with confirmed UC that were either seen at the outpatient clinic or as an inpatient at KAUH between January 2015 and December 2020. The primary outcome was to determine the proportion of patients with a history of recurrent UC flares, defined as experiencing more than one flare per year. A flare was defined as a documented worsening of a patient’s clinical picture in the form of deteriorating symptoms, coupled with an increase in inflammatory markers or confirmed increase in endoscopic disease activity (according to the Mayo endoscopic subscore (MES)). Secondary outcomes included testing for possible associations between smoking habits and UC flares and other potential predictors of flares.

Study instrument

Patients were asked to complete the study questionnaire. The questionnaire focused on smoking habits (cigarettes, shisha [also known as Hubbly Bubbly or Hookah], vape, and nicotine level), and included a section that addressed the patient’s perspectives on how smoking affected the course of their disease. Additional information was also collected from the patient medical records, including clinical and demographic data, anthropometric and radiologic findings, and relevant laboratory investigations. The frequency of admissions for UC flares was also recorded.

Ethical considerations

The study was approved by the research ethics committee at King Abdulaziz University Hospital, in Jeddah, KSA (#377-20), and was conducted in accordance with the Declaration of Helsinki.

Statistical analysis

Data were analyzed using the Statistical Package for the Social Sciences (SPSS) program, version 25 (IBM Corp., Armonk, NY). Qualitative data were presented as numbers and percentages, and chi-squared tests (χ^2^) were applied to examine possible relationships between categorical variables. Quantitative data were expressed as means and standard deviations (mean ± SD), and Mann-Whitney U tests and independent samples t-tests were incorporated for comparison according to the data distribution. Spearman’s correlation coefficient was used to test for possible correlations between variables. A P-value of <0.05 was considered statistically significant.

## Results

The IBDIS registry at KAUH was screened for eligible participants: 312 patients with UC were initially identified; 89 patients agreed to participate and were included in the study. The baseline characteristics of the study cohort: the mean age was 34.8 ± 16.81 years; 55.1% were males; 80.9% were of Saudi Arabian nationality; and 86.5% were obese, with a mean BMI of 42.02 ± 12.92 kg/m^2^. The mean number of residents in participant households was 5.34 ± 2.5. Family history of UC was reported by 25.8% of participants, and the mean duration of UC was 8.37 ± 6.56 years. According to MES, 20.2% had moderate inflammation (marked erythema, absent vascularity, friability, and erosions) documented on their first colonoscopy, while on the last colonoscopy about one-third of patients (33.7%) had moderate inflammation (marked erythema, absent vascularity, friability, and erosions). Approximately half of the participants (48.3%) were scored to have mild disease. Recurrent flares were reported by 48.3% of participants and 48.3% had 0-1 UC flares per year or were admitted to hospital due to severe UC flares. The mean number of admissions owing to a flare-up was 3.13 ± 2.65. Of the participants included, 89.9% and 66.3%, respectively, were previously treated with 5-ASA agents and immunomodulators, compared to 49.4% and 68.5% who currently received those same treatments (Table 1).

**Table 1:** Baseline characteristics of the study participants (n = 89). ASA: aminosalicylates, UC: ulcerative colitis, MES: Mayo endoscopic subscore.

Variable		No (%)
Age (mean ± SD)		34.8 ± 16.81
Gender	Female	40 (44.9)
	Male	49 (55.1)
Nationality	Not Saudi	17 (19.1)
	Saudi	72 (80.9)
Number of residents in the household		5.34 ± 2.5
BMI categories	Underweight	1 (1.1)
	Normal weight	4 (4.5)
	Overweight	7 (7.9)
	Obese	77 (86.5)
	BMI (mean ± SD)	42.02 ± 12.92
Family history of ulcerative colitis	No	66 (74.2)
	Yes	23 (25.8)
Duration of UC (mean ± SD)		8.37 ± 6.56
First colonoscopy finding (according to MES)	Mild inflammation	1 (1.1)
	Moderate inflammation	18 (20.2)
	Severe inflammation	9 (10.1)
	Aphthous ulcer	10 (11.2)
	Other	10 (11.2)
	Normal	8 (9)
	No colonoscopy done	33 (37.1)
Last colonoscopy finding (MES)	Mild inflammation	11 (12.4)
	Moderate inflammation	30 (33.7)
	Severe inflammation	5 (5.6)
	Aphthous ulcer	1 (1.1)
	Other	11 (12.4)
	Normal	16 (18)
	No colonoscopy done	15 (16.9)
Extent of disease	Intact (no involvement)	24 (26.9)
	Extensive UC (E3)	14 (15.7)
	Ulcerative proctitis (E1)	16 (18)
	Left-sided UC (E2)	35 (39.4)
Clinical disease activity index (Montreal classification UC severity)	Mild	43 (48.3)
	Moderate	13 (14.6)
	Severe	30 (33.7)
	None	3 (3.4)
Treatment history	5-ASA agents	59 (66.3)
	Immunomodulators	80 (89.9)
	Biologics	28 (31.5)
	Surgery	6 (6.7)
Current treatment	5-ASA agents	44 (49.4)
	Immunomodulators	61 (68.5)
	Biologics	30 (33.7)

Comparing patients with frequent flares with those who reported no flares, a higher rate of flare-up was observed in patients that were either under-or over-weight (P = 0.041), had a family history of UC (P = 0.013), had depression (P = 0.03), had fecal incontinence (P = 0.003), had iron deficiency anemia (P = 0.009), or malignancy (P = 0.039). A non-significant relationship was found between recurrent UC flares and previous or current treatment history (Table 2).

**Table 2:** A comparison between UC patients with and without a history of flares. N.B.: *Mann–Whitney U test. UC: ulcerative colitis, NSAID: nonsteroidal anti-inflammatory drug, ASA: aminosalicylates.

Variable		UC flares		χ^2^	P-value
		UC with recurrent flares No (%)	UC without recurrent flares No (%)		
Age (mean ± SD)		32.17 ± 15.58	37.62 ± 17.78	1.46*	0.144
Gender	Female	22 (55)	18 (45)	0.32	0.572
	Male	24 (49)	25 (51)		
Nationality	Not Saudi	7 (41.2)	10 (58.8)	0.92	0.3351
	Saudi	39 (54.2)	33 (45.8)		
BMI categories	Underweight	1 (100)	0 (0.0)	8.23	0.041
	Normal weight	2 (50)	2 (50)		
	Overweight	7 (100)	0 (0.0)		
	Obese	36 (46.8)	41 (53.2)		
History of NSAID use	Daily	1 (100)	0 (0.0)	4.88	0.30
	Frequently	5 (83.3)	1 (16.7)		
	Intermittent	1 (50)	1 (50)		
	Never	25 (44.6)	31 (55.4)		
	Rarely	14 (58.3)	10 (41.7)		
Family history of UC	No	29 (43.9)	37 (56.1)	6.13	0.013
	Yes	17 (73.9)	6 (26.1)		
Depression	Yes	9 (81.8)	2 (18.2)	4.56	0.033
Previous colon surgery	Yes	2 (28.6)	5 (71.4)	1.62	0.202
Appendectomy	Yes	1 (50)	1 (50)	0.002	0.962
Frequency of exercise (days per week)	1-2 per week	4 (23.5)	13 (76.5)	9.45	0.05
	3-4 per week	10 (76.9)	3 (23.1)		
	5-6 per week	5 (62.5)	3 (37.5)		
	Everyday	3 (42.9)	4 (57.1)		
	Never	24 (54.5)	20 (45.5)		
Severity	Mild	19 (44.2)	24 (55.8)	4.72	0.193
	Moderate	10 (76.9)	3 (23.1)		
	Severe	16 (53.3)	14 (46.7)		
	None	1 (33.3)	2 (66.7)		
Symptoms	Abdominal pain	41 (56.2)	32 (43.8)	3.26	0.071
	Vomiting	14 (66.7)	7 (33.3)	2.47	0.116
	Weight loss	38 (56.7)	29 (43.3)	2.74	0.097
	Dehydration	22 (55)	18 (45)	0.32	0.572
	Fecal incontinence	25 (71.4)	10 (28.6)	9	0.003
	Extra-intestinal manifestations	2 (66.7)	1 (33.3)	0.27	0.597
Complications	Iron deficiency anemia	23 (69.7)	10 (30.3)	6.81	0.009
	Osteoporosis	8 (80)	2 (20)	3.61	0.057
	Deep venous thrombosis	1 (100)	0 (0.0)	0.94	0.331
	Malignancy	12 (75)	4 (25)	4.24	0.039
Treatment history	5-ASA agents	30 (50.8)	29 (49.2)	0.04	0.824
	Immunomodulators	42 (52.5)	38 (47.5)	0.21	0.647
	Biologics	14 (50)	14 (50)	0.04	0.829
	Surgery	2 (33.3)	4 (66.7)	0.86	0.352
Current treatment	5-ASA agents	19 (43.2)	25 (56.9)	2.52	0.112
	Immunomodulators	35 (57.4)	26 (42.6)	2.51	0.113
	Biologics	16 (53.3)	14 (46.7)	0.04	0.824

Of the total cohort, for cigarette smoking, 8 patients (9%) were smokers and 13 (14.6%) were ex-smokers. For shisha smoking, 12 patients (13.5%) were current smokers and 6 were ex-smokers (6.7%). Only three patients (3.4 %) practiced vaping. Twenty patients (22.5%) perceived smoking as a cause of a large number of disease flares (Table 3).

**Table 3:** Distribution of studied patients according to smoking (cigarettes, shisha, vape) habits, and perception of how smoking affected the course of UC. UC: ulcerative colitis.

Variable		No. (%)
Cigarette smoking	Current smoker	8 (9)
	Ex-smoker	13 (14.6)
	Non-smoker	68 (79.4)
If ex-smoker for cigarettes, duration of cessation in years (mean ± SD)		5.53 ± 4.18
Number of years you are smoking cigarettes (mean ± SD)		9.81 ± 10.66
Shisha smoking	Current smoker	12 (13.5)
	Ex-smoker	6 (6.7)
	Non-smoker	71 (79.8)
If ex-smoker for shisha, duration of cessation in years (mean ± SD)		4.83 ± 4.16
Number of years you are smoking shisha (mean ± SD)		9.83 ± 12.69
How many heads per day (mean ± SD)		1.83 ± 0.78
History of vape	Current smoker	3 (3.4)
	Non-smoker	86 (96.6)
Do you feel that smoking affected the course of your disease?	I do not know	2 (2.2)
	Fewer flares	6 (6.7)
	More flares	20 (22.5)
	No change	3 (3.4)
	Not a smoker	58 (65.2)

There were no statistically significant differences found between recurrent UC flares and smoking habits of any type (cigarettes, shisha, vape), and between recurrent flares and feeling that smoking affected the course of UC (Table 4).

**Table 4:** Relationship between UC recurrent flares and smoking (cigarettes, shisha, vape) habits. N.B.: *Mann–Whitney U test; **Independent samples t-test. UC: ulcerative colitis.

Variable		UC flares		χ^2^	P-value
		Yes (%)	No (%)		
Cigarette smoking	Current smoker	3 (37.5)	5 (62.5)	3.79	0.15
	Ex-smoker	4 (30.8)	9 (69.2)		
	Non-smoker	39 (57.4)	29 (42.6)		
Number of years you are smoking cigarettes (mean ± SD)		46	43	0.59*	0.64
If ex-smoker for cigarettes, duration of cessation in years (mean ± SD)		6 ± 6.21	5.32 ± 3.4	0.25**	0.22
Shisha smoking	Current smoker	7 (58.3)	5 (42.7)	0.24	0.88
	Ex-smoker	3 (50)	3 (50)		
	Non-smoker	36 (50.7)	35 (49.3)		
Number of years you are smoking shisha (mean ± SD)		8.9 ± 11.16	11 ± 15.1	3.00*	0.32
Vape history	Current smoker	3 (100)	0 (0.0)	2.90	0.09
	Non-smoker	43 (50)	43 (50)		
How do you feel that smoking affected the course of your disease?	I do not know	1 (50)	1 (50)	1.72	0.78
	Fewer flares	2 (33.3)	4 (66.7)		
	More flares	9 (45)	11 (55)		
	No change	2 (66.7)	1 (33.3)		
	Not a smoker	32 (55.2)	26 (44.8)		

A binary logistic regression analysis has confirmed the presence of a positive family history of UC (OR = 5.3, P = 0.007) and fecal incontinence (OR = 4.7, P = 0.006) as independent predictors of UC disease flare-ups (Table 5).

**Table 5:** Logistic regression analysis of independent predictors of recurrent UC flare-ups. UC: ulcerative colitis.

Variable	B	P-value	Odds’ ratio (CI: 95%)
BMI categories	0.73	0.154	2.07 (2.98–1.98)
Family history of UC	1.66	0.007	5.30 (5.91–3.27)
Depression	1.35	0.158	3.86 (4.23–3.14)
Fecal incontinence as a symptom	0.54	0.006	4.70 (4.86–3.91)
Iron deficiency anemia as a complication	0.83	0.149	2.31 (2.65–2.01)
History of steroid use	0.83	0.198	2.29 (2.48–1.89)

## Discussion

UC is a chronic inflammatory bowel disease of unknown etiology and differentiates itself from CD by the exhibition of a continuous and symmetrical pattern of inflammation restricted to the colon [6]. The reported risk factors for IBD include male gender, smoking, history of appendectomy, various medications (contraceptive pills, non-steroidal anti-inflammatory drugs [NSAIDs], and antibiotics), dietary habits, stress, and family history of IBD [5]. The risk of UC increases among former smokers, with a milder disease being reported among current smokers than ex-smokers and non-smokers [9,11].

Our study found that hypertension, diabetes, and anxiety, although the most commonly reported comorbidities, were not associated significantly with recurrent flares, excepting depression. While most of the participants did not use NSAIDs (62.9 %), NSAIDs use was not associated significantly with recurrent flares. However, other studies have reported a significant association between NSAID medication, and an increased risk of IBD [13,14].

The present study found a significant association between an increased number of recurrent UC flares and being underweight, having a family history of UC, and suffering from depression. Fecal incontinence, iron deficiency anemia, and malignancy are associated significantly with increased UC activity in recurrent flares. Also, family history of UC and fecal incontinence were risk factors of having a greater number of recurrent UC flares. Bigeh et al. [15] reported that steroids intake increased the risk of atherosclerotic cardiovascular disease (ASCVD) in patients with IBD, which matches the findings of the present study.

Among our study participants, 9% were current cigarette smokers and 14.6% were ex-smokers. Also, 13.5% were current shisha smokers, and 3.4% vaped. Of all smokers, 22.5% felt that smoking negatively affected the disease course. Several studies assess the association between smoking habits and the development of UC [16-18]. It has been reported that heavier smokers were less likely to develop UC than lighter smokers, and current smokers had a milder disease compared to ex-smokers, which coheres with the results of the present study [16,18]. Another study reported that participants thought that smoking protected against flares and adverse progression of UC [16]. Nicotine has been linked with better outcomes of UC on the basis that it might attribute to the reduction of IL-1, IL-8, TNF α, and other pro-inflammatory cytokines levels [19]. AlQasrawi et al. [20] report a positive effect of smoking, and nicotine in general, on UC, attributing this finding to activation of the cholinergic anti-inflammatory pathway through the α7-nicotinic acetylcholine receptor, which causes a shift toward M2-macrophage polarization and results in downregulation of pro-inflammatory cytokines (IL-6 and tumor necrosis factor-alpha (TNF-α)) and upregulation of anti-inflammatory cytokine interleukin-10 (IL-10). A further study found complementary results to the present study: smokers with UC had a reduction in flares using steroids and a lower rate of colectomy compared to non-smokers with UC [21]. One study found that the risk of acquiring UC might be reduced among heavy smokers, based on dosage [22], and another study indicated that the risk of developing UC increased in ex-smokers [23].

Previous studies have noted that 84.1% of participants reported that they did not smoke, and a negative association was found between smoking and UC flares [18,24,25]. A study by Cope et al. [26] showed that non-smokers had a significantly lower glycoprotein production compared to controls while smoking patients had similar levels to controls. Smoking therefore may decrease the risk of UC by normalizing defective mucus production common with UC. This reflects the increased number of flares observed when smoking is discontinued and where patients are exposed to any of the risk factors associated with UC. Similarly, patients with UC that are heavy smokers have been found to be protected from recurrent heavy flares.

Soon et al. [27] reported a significant association between urban residency and developing UC, which is in line with our results; indeed, 95.5% of our study population lived in urban areas. Psychological depression has been associated with increased IBD flares, which corresponds with our results [28]. The association between family history of IBD and UC development has been reported in a number of studies [29-31]. It has been shown that 8-14% of patients with UC had a family history of UC [32], and the risk for developing UC among offspring is higher if both parents are affected [33].

The present study may be limited by its small sample size and retrospective design. A larger population is required to more definitively illustrate associations between smoking and UC.

## Conclusions

Fecal incontinence and a family history of UC were found to be associated with an increased risk of developing recurrent flares. There was no clear association found between smoking and recurrent UC flares. Further prospective studies with larger sample sizes are required to better evaluate the relationship between smoking and the frequency and severity of UC flares.

## Appendices

In the data below, Part A was gathered from the patient, and Part B was collected from the medical records of King Abdulaziz University Hospital.

Part A (From the patient)

Age (short answer)

Gender

-       Male

-       Female

Nationality

-       Saudi

-       Not Saudi

Height in Meters (short answer)

Weight in Kilograms (short answer)

Body mass index (short answer) (calculated by excel)

-       Underweight

-       Normal weight

-       Overweight

-       Obese

Number of residence in the household (short answer)

Family history of ulcerative colitis

-       Yes

-       No

Depression

-       Yes

-       No

Previous colon Surgery

-       Yes

-       No

Appendectomy

-       Yes

-       No

Blood in stool

-       Yes

-       No

Abdominal pain

-       Yes

-       No

Vomiting

-       Yes

-       No

Weight loss

-       Yes

-       No

Dehydration

-       Yes

-       No

Fecal incontinence

-       Yes

-       No

Iron deficiency anemia

-       Yes

-       No

Osteoporosis

-       Yes

-       No

Deep venous thrombosis

-       Yes

-       No

Malignancy

-       Yes

-       No

History of NSAIDs use

-       Daily

-       Frequently

-       Intermittent

-       Rarely

-       Never

Frequency of exercise (days per week)

-       1-2 per week

-       3-4 per week

-       5-6 per week

-       Everyday

-       Never

Treatment History

-       5-ASA agents

-       Immunomodulators

-       Biologics

-       Surgery

Current treatment

-       5-ASA agents

-       Immunomodulators

-       Biologics

Cigarette smoking

-       Current smoker

-       Ex-smoker

-       Non-smoker

If ex-smoker for cigarettes, duration of cessation in years (short answer)

Number of years you are smoking cigarettes (short answer)

Shisha smoking

-       Current smoker

-       Ex-smoker

-       Non-smoker

If ex-smoker for shisha, duration of cessation in years (short answer)

Number of years you are smoking shisha (short answer)

How many heads per day (short answer)

History of Vape

-       Current smoker

-       Ex-smoker

-       Non-smoker

Do you feel that smoking affected the course of your disease?

-       I don't know

-       Less flares

-       More flares

-       No change

-       Not a smoker

Part B (From the medical records)

First colonoscopy finding (according to Mayo Endoscopic Score)

-       Mild inflammation

-       Moderate inflammation

-       Severe inflammation

-       Apthous ulcer

-       Other

-       Normal

-       No colonoscopy done

Last colonoscopy finding (according to Mayo Endoscopic Score)

-       Mild inflammation

-       Moderate inflammation

-       Severe inflammation

-       Apthous ulcer

-       Other

-       Normal

-       No colonoscopy done

Extent of the disease

-       Intact (no involvement)

-       Ulcerative proctitis (E1)

-       Left-sided UC (E2)

-       Extensive UC (E3)

Clinical disease activity index (Montreal classification UC severity)

-       Mild

-       Moderate

-       Severe

-       None
